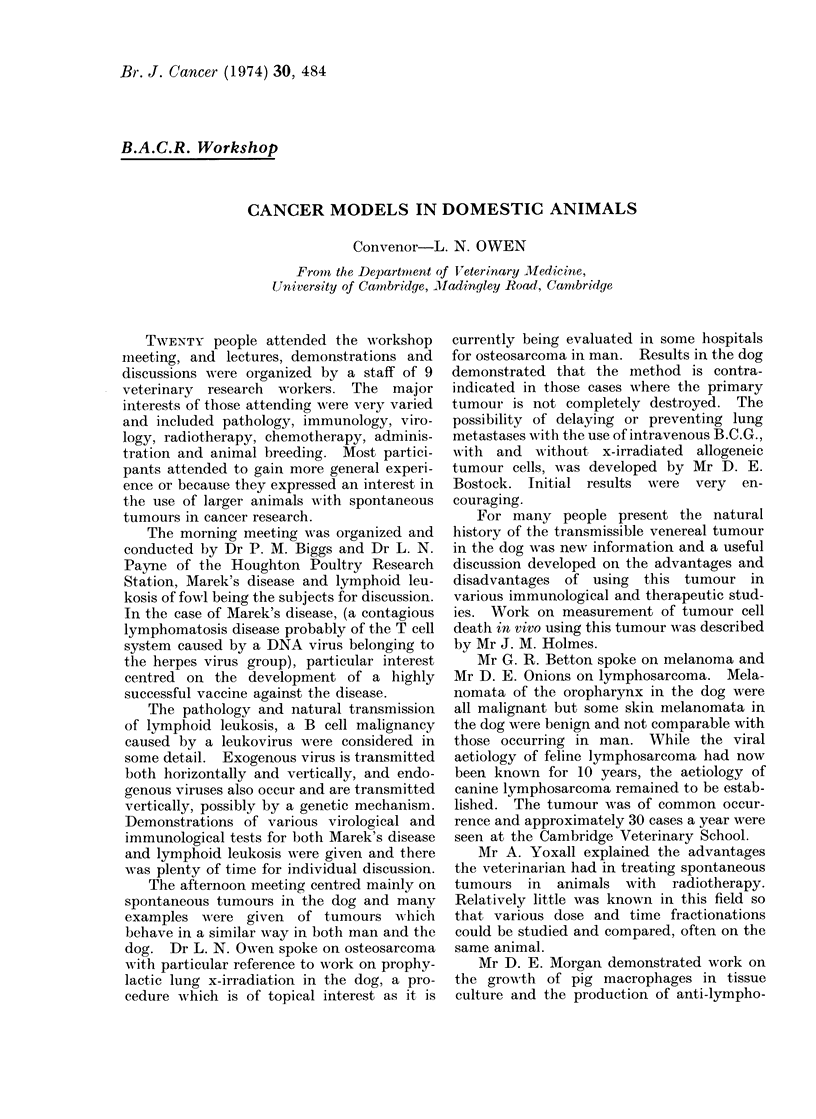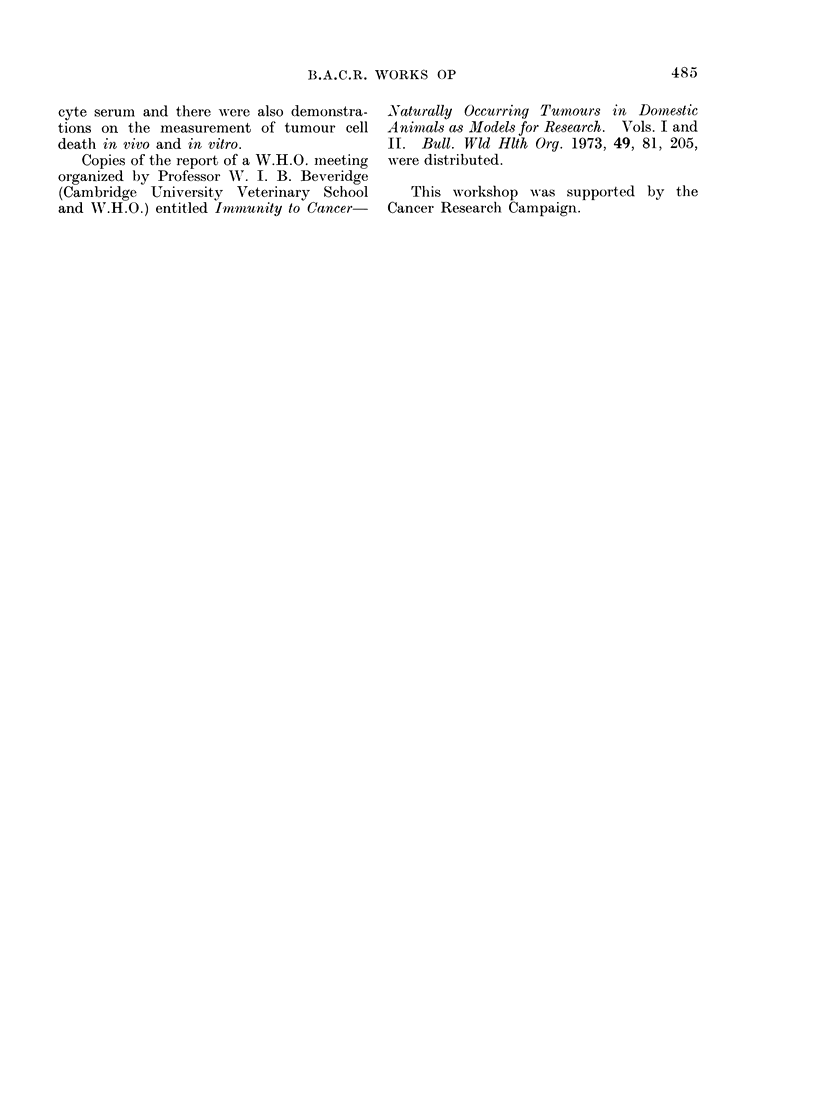# Cancer Models in Domestic Animals

**Published:** 1974-11

**Authors:** 


Br. J. Cancer (1974) 30, 484

### B.A.C.R. Workshop

### CANCER MODELS IN DOMESTIC ANIMALS

Convenor—L. N. OWEN

*From the Department of Veterinary Medicine, University of Cambridge, Madingley Road, Cambridge*

TWENTY people attended the workshop meeting, and lectures, demonstrations and discussions were organized by a staff of 9 veterinary research workers. The major interests of those attending were very varied and included pathology, immunology, virology, radiotherapy, chemotherapy, administration and animal breeding. Most participants attended to gain more general experience or because they expressed an interest in the use of larger animals with spontaneous tumours in cancer research.

The morning meeting was organized and conducted by Dr P. M. Biggs and Dr L. N. Payne of the Houghton Poultry Research Station, Marek's disease and lymphoid leukosis of fowl being the subjects for discussion. In the case of Marek's disease, (a contagious lymphomatosis disease probably of the T cell system caused by a DNA virus belonging to the herpes virus group), particular interest centred on the development of a highly successful vaccine against the disease.

The pathology and natural transmission of lymphoid leukosis, a B cell malignancy caused by a leukovirus were considered in some detail. Exogenous virus is transmitted both horizontally and vertically, and endogenous viruses also occur and are transmitted vertically, possibly by a genetic mechanism. Demonstrations of various virological and immunological tests for both Marek's disease and lymphoid leukosis were given and there was plenty of time for individual discussion.

The afternoon meeting centred mainly on spontaneous tumours in the dog and many examples were given of tumours which behave in a similar way in both man and the dog. Dr L. N. Owen spoke on osteosarcoma with particular reference to work on prophylactic lung x-irradiation in the dog, a procedure which is of topical interest as it is currently being evaluated in some hospitals for osteosarcoma in man. Results in the dog demonstrated that the method is contraindicated in those cases where the primary tumour is not completely destroyed. The possibility of delaying or preventing lung metastases with the use of intravenous B.C.G., with and without x-irradiated allogeneic tumour cells, was developed by Mr D. E. Bostock. Initial results were very encouraging.

For many people present the natural history of the transmissible venereal tumour in the dog was new information and a useful discussion developed on the advantages and disadvantages of using this tumour in various immunological and therapeutic studies. Work on measurement of tumour cell death *in vivo* using this tumour was described by Mr J. M. Holmes.

Mr G. R. Betton spoke on melanoma and Mr D. E. Onions on lymphosarcoma. Melanomata of the oropharynx in the dog were all malignant but some skin melanomata in the dog were benign and not comparable with those occurring in man. While the viral aetiology of feline lymphosarcoma had now been known for 10 years, the aetiology of canine lymphosarcoma remained to be established. The tumour was of common occurrence and approximately 30 cases a year were seen at the Cambridge Veterinary School.

Mr A. Yoxall explained the advantages the veterinarian had in treating spontaneous tumours in animals with radiotherapy. Relatively little was known in this field so that various dose and time fractionations could be studied and compared, often on the same animal.

Mr D. E. Morgan demonstrated work on the growth of pig macrophages in tissue culture and the production of anti-lymphocyte serum and there were also demonstrations on the measurement of tumour cell death *in vivo* and *in vitro.*

Copies of the report of a W.H.O. meeting organized by Professor W. I. B. Beveridge (Cambridge University Veterinary School and W.H.O.) entitled *Immunity to Cancer—Naturally Occurring Tumours in Domestic Animals as Models for Research.* Vols. I and II. *Bull. Wld Hlth Org.* 1973, **49**, 81, 205, were distributed.

This workshop was supported by the Cancer Research Campaign.